# Correlation Between Hypophosphatemia and Hyperventilation in Critically Ill Patients: Causes, Clinical Manifestations, and Management Strategies

**DOI:** 10.3390/biomedicines13102382

**Published:** 2025-09-28

**Authors:** Nicola Sinatra, Giuseppe Cuttone, Giulio Geraci, Caterina Carollo, Michele Fici, Tarek Senussi Testa, Luigi La Via

**Affiliations:** 1 Nephrology and Dialysis Unit, “Paolo Borsellino” Hospital, 91025 Marsala, Italy; sinatra.nicola@libero.it (N.S.); mfici21@gmail.com (M.F.); 2 Trauma Center Unit, “Villa Sofia-Cervello” Hospital, 90146 Palermo, Italy; giuseppe.cuttone@hotmail.it; 3 Faculty of Medicine and Surgery, Kore University, 94100 Enna, Italy; giulio.geraci@unikore.it; 4 Unit of Nephrology and Dialysis, Hypertension Excellence Centre, Department of Health Promotion, Mother and Child Care, Internal Medicine and Medical Specialties (PROMISE), University of Palermo, 90133 Palermo, Italy; caterina.carollo@unipa.it; 5 Department of Cardiac Anesthesia and Intensive Care, Cardiovascular Network, IRCCS Policlinico San Martino Hospital, 16132 Genova, Italy; t.senussi@hotmail.it; 6 Department of Anesthesia and Intensive Care 1, University Hospital Policlinico “G. Rodolico-San Marco”, 95123 Catania, Italy

**Keywords:** hypophosphatemia, hyperventilation, 2,3-diphosphoglycerate, ICU, mechanical ventilation

## Abstract

Hypophosphatemia, defined as serum phosphate levels below 2.5 mg/dL, is a common yet underrecognized electrolyte disturbance in critically ill patients, with prevalence estimates reaching up to 80%. This review explores the intricate bidirectional relationship between hypophosphatemia and hyperventilation, emphasizing its profound implications for respiratory function and critical care management. Hypophosphatemia impairs oxygen delivery by depleting 2,3-diphosphoglycerate (2,3-DPG), disrupts central respiratory drive, and weakens respiratory muscles, leading to hyperventilation, ventilatory failure, and prolonged mechanical ventilation. Conversely, hyperventilation exacerbates hypophosphatemia through respiratory alkalosis, triggering intracellular phosphate shifts and metabolic cascades that rapidly deplete serum levels. This cycle creates significant challenges for ventilator weaning and increases morbidity and mortality. Underlying mechanisms include impaired ATP synthesis, altered chemoreceptor sensitivity, and systemic inflammatory responses. Hypophosphatemia-induced hyperventilation manifests as unexplained tachypnea and respiratory alkalosis, often misdiagnosed as anxiety or pain, while hyperventilation-induced hypophosphatemia contributes to diaphragmatic dysfunction and poor ventilatory performance. Common precipitating factors include refeeding syndrome, diabetic ketoacidosis, continuous renal replacement therapy, and malnutrition. Complications extend beyond respiratory dysfunction to include cardiac depression, immune dysfunction, prolonged ICU stays, and increased healthcare costs. Current diagnostic approaches rely on serum phosphate measurements, which poorly reflect total body stores due to significant intracellular shifts. Emerging biomarkers such as fibroblast growth factor 23 (FGF23) and advanced monitoring technologies, including continuous phosphate tracking, may enhance recognition. Treatment strategies emphasize targeted phosphate repletion based on severity, with intravenous supplementation and ventilatory support tailored to minimize complications. Preventive measures, including risk stratification, prophylactic supplementation, and ventilator management, are critical for high-risk populations. Despite advances, knowledge gaps persist in optimizing monitoring and repletion protocols, understanding genetic variations, and identifying ideal phosphate targets for improved respiratory outcomes. This review provides a comprehensive framework for recognizing and managing hypophosphatemia’s impact on respiratory dysfunction in critically ill patients. Adopting evidence-based interventions and leveraging emerging technologies can significantly improve clinical outcomes, reduce ICU complications, and enhance recovery in this vulnerable population.

## 1. Introduction: The Overlooked Connection Between Phosphate Homeostasis and Respiratory Drive

Hypophosphatemia, defined as serum phosphate below 2.5 mg/dL, is a frequently encountered yet underrecognized electrolyte disturbance in critically ill patients, with prevalence ranging from 20–80% [1,2]. Despite its clinical significance, the relationship between phosphate depletion and respiratory dysfunction remains inadequately appreciated in intensive care practice [3]. While hypophosphatemia can cause respiratory complications, hyperventilation itself induces hypophosphatemia through respiratory alkalosis, triggering intracellular phosphate shifts that can profoundly deplete serum levels within hours [4,5]. Phosphate serves essential roles as a component of ATP and 2,3-diphosphoglycerate (2,3-DPG), crucial for cellular energy and oxygen delivery [6]. Phosphate depletion profoundly impacts central respiratory drive and peripheral muscle function, causing unexplained hyperventilation, difficult ventilator weaning, and respiratory muscle weakness [7,8]. First described in the 1970s, hypophosphatemia-induced hyperventilation results from multiple mechanisms including impaired oxygen delivery through decreased 2,3-DPG, altered chemoreceptor sensitivity, and intracellular pH changes [4,9,10]. Hyperventilation-induced respiratory alkalosis specifically increases intracellular pH, stimulating phosphofructokinase activity and driving phosphate into cells for ATP synthesis [8,11].

In critical care, hypophosphatemia develops through decreased intake, increased cellular uptake, enhanced renal losses, and redistribution, with common precipitants including refeeding syndrome, diabetic ketoacidosis treatment, continuous renal replacement therapy, and phosphate-binding antacids [12,13,14]. These respiratory consequences become particularly relevant during recovery when ventilator weaning may be hampered [15]. Clinical recognition is challenging as hyperventilation with compensated respiratory alkalosis is often misattributed to anxiety or pain, leading to inappropriate interventions [5,16]. Individual variation exists in the phosphate threshold affecting respiratory function [17].

Recent advances have revealed novel phosphate-sensing pathways in chemoreceptor cells and respiratory neurons [18,19]. Chronic hypophosphatemia may cause persistent adaptive changes in respiratory control [20]. The economic and clinical burden is substantial, with severe hypophosphatemia associated with prolonged mechanical ventilation, increased ventilator-associated pneumonia, extended ICU stays, and healthcare costs exceeding $50,000 per patient [21,22,23].

Significant knowledge gaps remain regarding monitoring strategies and repletion protocols. Current practice relies on intermittent serum measurements that poorly reflect intracellular stores [24]. Optimal repletion strategies remain controversial due to potential complications [25,26]. This review synthesizes current knowledge on pathophysiological mechanisms, assessment tools, and management strategies to provide a practical framework for recognizing and managing this overlooked cause of respiratory dysfunction in critically ill patients.

## 2. Phosphate Physiology and Regulation in Critical Illness

**Figure 1:**
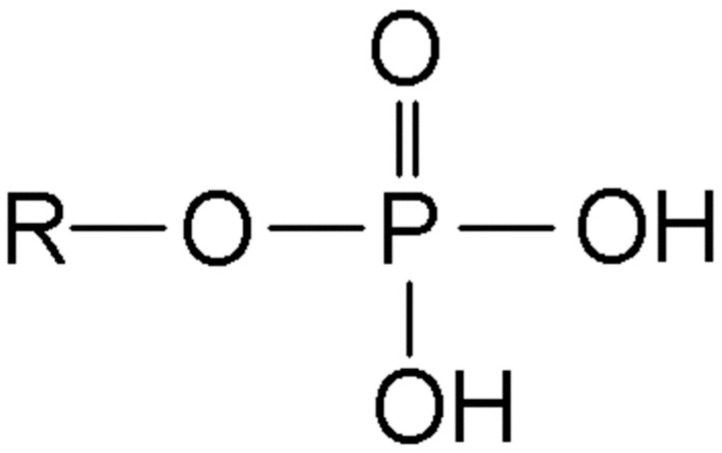
Chemical structure of phosphate.

Phosphate (Figure 1) represents the most abundant intracellular anion and plays crucial roles in cellular metabolism, energy storage, and signal transduction. In healthy adults, total body phosphate approximates 700 g, with 85% stored in bone as hydroxyapatite, 14% in soft tissues, and less than 1% in extracellular fluid [27]. The normal serum phosphate concentration ranges from 2.5 to 4.5 mg/dL (0.81–1.45 mmol/L), representing only 0.1% of total body stores, which explains why serum levels poorly reflect total body phosphate status [28].

Phosphate homeostasis is maintained through a complex interplay between intestinal absorption, renal excretion, and bone metabolism. Daily phosphate intake typically ranges from 1000–1500 mg, with 60–70% absorbed primarily in the jejunum through both passive paracellular diffusion and active transcellular transport mediated by sodium-phosphate cotransporters [29]. The kidneys filter approximately 4–6 g of phosphate daily, with 80–90% reabsorbed in the proximal tubule via NaPi-IIa and NaPi-IIc transporters [30].

Three primary hormones regulate phosphate balance: parathyroid hormone (PTH), FGF23, and 1,25-dihydroxyvitamin D (calcitriol). PTH and FGF23 promote phosphaturia by downregulating renal sodium-phosphate cotransporters, while calcitriol enhances intestinal absorption [31]. FGF23, produced by osteocytes in response to elevated phosphate levels, has emerged as a master regulator of phosphate homeostasis, with levels increasing within hours of phosphate loading [32].

Phosphate also plays a crucial role in acid-base homeostasis through the Stewart-Fencl physicochemical approach, where it functions as a weak acid contributing to the strong ion difference and influencing pH regulation. As a non-volatile weak acid with a pKa of 6.8, phosphate significantly impacts the apparent strong ion difference (SIDa) and the effective strong ion difference (SIDe), with hypophosphatemia reducing buffering capacity and potentially masking underlying metabolic acidosis [33]. The phosphate–calcium system serves as an important buffer for metabolic disturbances, particularly metabolic acidosis frequently encountered in critically ill patients, with each 1 mg/dL decrease in serum phosphate reducing buffering capacity by approximately 0.5 mEq/L. In critically ill patients with metabolic acidosis, the mobilization of phosphate from bone and intracellular stores not only serves as a buffering mechanism but may also contribute to the development of hypophosphatemia, creating a complex interplay between acid-base status and phosphate homeostasis [34].

Critical illness profoundly disrupts normal phosphate regulation through multiple mechanisms. Systemic inflammation triggers the release of pro-inflammatory cytokines including tumor necrosis factor-α, interleukin-1β, and interleukin-6, which directly suppress renal phosphate reabsorption and increase cellular uptake [24]. Additionally, critical illness-associated stress hormones, particularly catecholamines and cortisol, promote intracellular phosphate shifts through enhanced glucose uptake and glycolysis [35].

The acute phase response characteristic of critical illness further complicates phosphate homeostasis. Increased hepatic glucose production, insulin resistance, and enhanced cellular metabolism create substantial phosphate demands for ATP synthesis and intermediary metabolism [36]. Simultaneously, gastrointestinal dysfunction, common in critically ill patients, impairs phosphate absorption, while acute kidney injury, present in up to 50% of ICU patients, disrupts normal renal phosphate handling [37,38].

Medications commonly used in critical care significantly impact phosphate balance. Continuous renal replacement therapy causes substantial phosphate losses, with clearance rates of 30–50 mL/min leading to daily losses exceeding normal intake [14]. Diuretics, particularly loop and thiazide diuretics, enhance renal phosphate excretion, while aluminum- and magnesium-containing antacids bind intestinal phosphate, preventing absorption [39]. Insulin administration, whether for glycemic control or hyperkalemia treatment, drives phosphate intracellularly, potentially precipitating severe hypophosphatemia [40].

The refeeding syndrome represents a particularly important cause of hypophosphatemia in critical illness. During starvation, total body phosphate depletes while serum levels remain normal due to release from intracellular stores. Upon refeeding, insulin-stimulated cellular uptake rapidly depletes serum phosphate, potentially causing life-threatening complications [13]. Risk factors include malnutrition, alcoholism, anorexia nervosa, and prolonged fasting, conditions frequently encountered in critically ill patients [41].

Recent research has identified novel phosphate-sensing mechanisms that may influence critical illness outcomes. The phosphate transporter PiT1 functions as a phosphate sensor, triggering intracellular signaling cascades that regulate cellular metabolism and survival [18]. Additionally, extracellular phosphate directly modulates inflammatory responses, with hypophosphatemia associated with impaired neutrophil function, decreased macrophage cytokine production, and increased susceptibility to sepsis [42].

Understanding phosphate physiology in critical illness requires recognizing the dynamic nature of phosphate distribution and regulation. Serum phosphate levels fluctuate significantly throughout the day and in response to various stimuli, necessitating careful interpretation in the context of clinical status, medications, and ongoing therapies [43]. As our understanding of phosphate biology expands, targeted therapeutic strategies addressing specific disruptions in phosphate homeostasis may improve outcomes in critically ill patients.

## 3. Bidirectional Pathophysiological Mechanisms Between Hypophosphatemia and Hyperventilation

The relationship between hypophosphatemia and hyperventilation represents a complex bidirectional pathophysiological process where each condition can precipitate and perpetuate the other, creating a potentially dangerous cycle in critically ill patients. Understanding these interconnected mechanisms is crucial for effective clinical management.

### 3.1. Hypophosphatemia Leading to Hyperventilation

Hypophosphatemia triggers hyperventilation primarily through impaired oxygen delivery. The most established mechanism involves depletion of 2,3-DPG in red blood cells. Since phosphate serves as the essential substrate for 2,3-DPG synthesis, severe hypophosphatemia leads to profound 2,3-DPG depletion within 24–48 h [44]. This depletion causes a leftward shift in the oxygen–hemoglobin dissociation curve, with P50 values decreasing by 4–8 mmHg in patients with phosphate levels below 1.0 mg/dL, significantly compromising oxygen unloading at tissue level [45,46]. The resulting tissue hypoxia, despite normal arterial oxygen content, stimulates peripheral chemoreceptors, particularly carotid bodies, triggering compensatory hyperventilation as an ineffective attempt to maintain tissue oxygenation [47].

Direct effects on respiratory control centers compound this response. Phosphate depletion enhances brainstem chemosensitivity to carbon dioxide and pH, lowering the ventilatory stimulation threshold [48]. This manifests as exaggerated ventilatory responses to normal physiological stimuli. Neurophysiological alterations include disrupted glutamatergic and GABAergic signaling pathways in respiratory centers, while impaired neuronal ATP production affects ATP-sensitive potassium channels that modulate respiratory neuron excitability [49].

Paradoxically, hypophosphatemia causes intracellular acidosis despite systemic alkalosis from hyperventilation. Phosphate depletion impairs cellular buffering capacity, leading to intracellular hydrogen ion accumulation that directly stimulates respiratory centers, perpetuating hyperventilation [50]. Additionally, compromised ATP synthesis creates an energy crisis particularly affecting metabolically active tissues including respiratory muscles and chemoreceptor cells [51]. 31P magnetic resonance spectroscopy studies demonstrate decreased phosphocreatine/inorganic phosphate ratios, confirming impaired cellular energetics [52].

Respiratory muscle dysfunction, while not directly causing hyperventilation, contributes to altered breathing patterns. Phosphate depletion impairs diaphragmatic contractility and endurance, leading to rapid shallow breathing as patients attempt to maintain minute ventilation with weakened muscles [5,7]. Recent research has identified additional mechanisms including altered calcium handling, impaired excitation-contraction coupling, and reduced mitochondrial oxidative capacity in respiratory muscles during phosphate depletion [53].

### 3.2. Hyperventilation Inducing Hypophosphatemia

Conversely, hyperventilation rapidly induces hypophosphatemia through respiratory alkalosis-triggered metabolic cascades. Excessive carbon dioxide elimination raises blood pH, initiating intracellular changes that can deplete serum phosphate within hours, particularly in patients with preexisting depletion [4,48].

The primary mechanism involves pH-mediated glycolytic activation. Respiratory alkalosis increases intracellular pH, stimulating phosphofructokinase, glycolysis’s rate-limiting enzyme [11]. Enhanced glycolytic flux consumes inorganic phosphate to produce phosphorylated intermediates including glucose-6-phosphate, fructose-6-phosphate, and fructose-1,6-bisphosphate, sequestering substantial phosphate amounts intracellularly [54]. The increased pH simultaneously promotes sugar phosphate formation and glucose metabolite phosphorylation, effectively trapping phosphate within cells [55]. 31 P-nuclear magnetic resonance spectroscopy confirms increased intracellular phosphomonoester concentrations during respiratory alkalosis [56]. A predictable relationship exists between pH and phosphate shifts: each 0.1 unit pH increase typically decreases serum phosphate by approximately 0.5 mg/dL through intracellular redistribution [50]. This relationship remains consistent across various alkalosis causes, though depletion magnitude depends on baseline stores and hyperventilation duration [57].

Beyond glycolysis, alkaline pH stimulates the pentose phosphate pathway, generating phosphorylated intermediates for nucleotide synthesis [58]. Enhanced ATP synthesis consumes inorganic phosphate for high-energy bond formation. These combined effects rapidly deplete accessible phosphate pools during sustained hyperventilation [44].

Hormonal responses amplify these shifts. Respiratory alkalosis stimulates insulin release independent of glycemia, promoting cellular glucose and phosphate uptake [59]. Concurrent catecholamine release from critical illness stress enhances glycogenolysis and glucose utilization, increasing phosphate demands [60].

Renal phosphate handling also changes, though less prominently than cellular shifts. Acute alkalemia initially reduces phosphate excretion through enhanced proximal tubular reabsorption [61], but prolonged alkalosis may impair conservation through altered sodium-phosphate cotransporter expression and function [62].

### 3.3. Clinical Implications

Several factors determine severity: baseline phosphate stores, with malnourished patients at highest risk [8]; degree of hyperventilation, with minute ventilation exceeding 15 L/min or PaCO_2_ below 25 mmHg substantially increasing risk [7]; and critical illness factors including systemic inflammation enhancing cellular phosphate uptake through cytokine-mediated mechanisms [24].

Common ICU interventions including mechanical ventilation, sedation withdrawal, and neurological injuries may precipitate hyperventilation [63]. Understanding these bidirectional mechanisms has crucial clinical implications. The rapid onset necessitates vigilant monitoring in at-risk patients. Recognition that serum levels poorly reflect total body phosphate status is essential, as intracellular shifts occur despite adequate stores [64].

Prolonged immobilization and neuromuscular blocking agents (NMBA) significantly affect phosphate homeostasis in critically ill patients. Immobilization leads to decreased bone turnover and altered phosphate metabolism, with studies showing 15–20% reduction in serum phosphate levels after 7 days of bed rest [65]. Continuous NMBA administration exacerbates muscle catabolism and phosphate release while simultaneously impairing cellular uptake mechanisms [66]. Early mobilization and physical therapy interventions help maintain phosphate homeostasis by preserving muscle mass and promoting normal bone-muscle phosphate exchange, with mobilized patients showing 30% lower incidence of severe hypophosphatemia compared to immobilized controls [67].

This knowledge guides prevention strategies and treatment approaches tailored to underlying mechanistic disturbances, emphasizing the importance of addressing both hypophosphatemia and hyperventilation simultaneously to break this potentially dangerous cycle.

## 4. Clinical Manifestations and Recognition of Hypophosphatemia and Hyperventilation Syndrome

The bidirectional relationship between hypophosphatemia and hyperventilation produces a complex clinical syndrome with diverse manifestations (Table 1) that often escape recognition in critically ill patients. Understanding these presentations and diagnostic approaches is essential for timely intervention.

### 4.1. Respiratory Manifestations

The hallmark of hypophosphatemia-induced hyperventilation is unexplained tachypnea with increased minute ventilation, typically developing when serum phosphate falls below 1.5 mg/dL, though susceptible individuals may exhibit symptoms at higher levels [9]. Patients demonstrate respiratory rates of 25–40 breaths per minute with reduced tidal volumes, creating a rapid, shallow breathing pattern persisting despite normal arterial oxygen saturation [20]. This invariably produces compensated respiratory alkalosis, with arterial blood gases revealing pH 7.45–7.55, PaCO_2_ 20–30 mmHg, and appropriately reduced bicarbonate [4]. Unlike primary respiratory alkalosis, patients rarely experience perioral paresthesias or carpopedal spasm, as concurrent hypophosphatemia paradoxically protects against alkalosis-induced hypocalcemia [68].

Conversely, when hyperventilation causes hypophosphatemia, respiratory muscle weakness becomes the predominant concern. Diaphragmatic dysfunction can precipitate ventilatory failure and complicate weaning from mechanical ventilation [7].

Patients exhibit decreased maximal inspiratory pressure, reduced transdiaphragmatic pressure, and impaired response to hypercapnic drive, with up to 50% developing proximal muscle weakness affecting cough effort and general strength [8].

### 4.2. Neuromuscular and Neurological Features

Neurological manifestations occur frequently with phosphate levels below 1.5 mg/dL, including altered mental status ranging from irritability and confusion to delirium and coma [69]. These symptoms often precede respiratory changes and may be mistakenly attributed to other causes. Seizures, though rare, may occur with severe depletion. Peripheral neuropathy presenting as paresthesias, hyperesthesia, and tremor has been reported, with central pontine myelinolysis documented in severe cases [70].

Rhabdomyolysis may occur with phosphate levels below 0.5 mg/dL, evidenced by elevated creatine kinase and myoglobinuria [60]. Peripheral muscle weakness manifests as proximal myopathy and difficulty with ambulation [9].

### 4.3. Hematological and Cardiac Abnormalities

Hematological abnormalities reflect impaired cellular metabolism. Hemolytic anemia results from decreased erythrocyte ATP content and 2,3-diphosphoglycerate depletion, leading to increased red cell rigidity and splenic sequestration [71]. Leukocyte dysfunction manifests as impaired chemotaxis, phagocytosis, and bactericidal activity, increasing infection susceptibility [45]. Platelet dysfunction may cause prolonged bleeding time, though clinically significant bleeding remains uncommon [42].

Cardiac manifestations include reversible myocardial depression with decreased contractility and reduced cardiac output [52]. Electrocardiographic changes may include ST-segment depression, T-wave flattening, and QT prolongation. Severe hypophosphatemia can precipitate congestive heart failure, particularly with underlying cardiac disease, though ventricular arrhythmias are relatively rare [72].

### 4.4. Metabolic Consequences

Metabolic disturbances encompass insulin resistance and impaired glucose metabolism from decreased ATP availability for insulin signaling and glucose transport [49]. Metabolic acidosis may paradoxically occur despite initiating respiratory alkalosis, resulting from impaired renal acid excretion and increased organic acid production [50].

In acute liver failure, hypophosphatemia represents a particularly ominous finding with unique pathophysiological implications. The liver plays a central role in phosphate metabolism through synthesis of phosphate-binding proteins and regulation of vitamin D metabolism. Severe hypophosphatemia (<1.0 mg/dL) occurs in 45–65% of acute liver failure patients and independently predicts poor outcomes, with mortality rates exceeding 80% when phosphate levels fall below 0.5 mg/dL [73]. The mechanism involves massive hepatocyte necrosis releasing intracellular phosphate initially, followed by profound depletion due to impaired hepatic regeneration requiring substantial phosphate for DNA synthesis and cellular proliferation. Additionally, acute liver failure impairs the conversion of 25-hydroxyvitamin D to its active form, further compromising phosphate homeostasis [74].

### 4.5. Diagnostic Approach

Recognition requires high clinical suspicion, as presentation mimics conditions including anxiety, pain, sepsis, pulmonary embolism, and central neurogenic hyperventilation [51]. Key distinguishing features include a temporal relationship between phosphate depletion and hyperventilation onset, absence of hypoxemia or significant lung pathology, and failure to respond to conventional interventions [53].

Diagnostic evaluation begins with serum phosphate measurement, though levels may not accurately reflect total body stores due to transcellular shifts [5]. Serial measurements capture the nadir, as levels fluctuate with ventilation changes, feeding status, and interventions [6]. Arterial blood gas analysis revealing respiratory alkalosis (pH > 7.45, PaCO_2_ < 35 mmHg) with hypophosphatemia supports hyperventilation-induced etiology [48]. Normal alveolar-arterial oxygen gradient distinguishes this from primary pulmonary pathology [46].

Additional evaluation should include comprehensive metabolic panel, magnesium, calcium, and vitamin D levels [40]. Continuous pulse oximetry and end-tidal CO_2_ monitoring reveal persistent hyperventilation with low ETCO_2_ despite normal oxygen saturation [75].

### 4.6. Risk Stratification

High-risk populations include patients with malnutrition undergoing refeeding, those receiving continuous renal replacement therapy, diabetic ketoacidosis during treatment, chronic alcoholism, and prolonged mechanical ventilation [1,76]. The syndrome frequently develops 48–72 h after initiating nutrition in previously starved patients or following insulin administration [12,77]. Response to phosphate repletion provides diagnostic confirmation, with hyperventilation typically resolving within 24–48 h of achieving normal levels [78].

## 5. Diagnostic Approaches, Severity Assessment, and Risk Stratification for Hypophosphatemia-Hyperventilation Syndrome

### 5.1. Laboratory Diagnosis and Monitoring

Diagnosis of hypophosphatemia relies primarily on serum phosphate measurement, though this represents only 1% of total body stores and may not accurately reflect intracellular depletion [79]. Normal serum phosphate ranges from 2.5–4.5 mg/dL, with hypophosphatemia classified as mild (2.0–2.5 mg/dL), moderate (1.0–2.0 mg/dL), or severe (<1.0 mg/dL) [4]. Symptoms of hyperventilation may occur at any level below 2.0 mg/dL, necessitating clinical correlation rather than relying solely on absolute values [5].

Timing significantly affects results, as phosphate levels exhibit diurnal variation with nadir values in early morning and peaks in late afternoon [80]. Respiratory alkalosis from any cause can acutely lower serum phosphate by 0.5–1.0 mg/dL through intracellular shifts, potentially masking true depletion severity [48]. Serial measurements over 24–48 h provide more accurate assessment than isolated values.

### 5.2. Comprehensive Metabolic Assessment

Comprehensive evaluation includes measurement of related electrolytes, as concurrent hypocalcemia, hypomagnesemia, and hypokalemia frequently accompany hypophosphatemia and may exacerbate clinical manifestations [73]. Arterial blood gas analysis revealing unexplained respiratory alkalosis (pH > 7.45, PaCO_2_ < 35 mmHg) with hypophosphatemia strongly suggests phosphate-induced hyperventilation [20].

Red blood cell 2,3-DPG levels provide direct evidence of impaired oxygen delivery, with values below 3 μmol/g hemoglobin indicating significant depletion [44]. When unavailable, calculating P50 from arterial blood gas co-oximetry can demonstrate the leftward shift in oxygen–hemoglobin dissociation curve characteristic of 2,3-DPG depletion [81].

### 5.3. Severity Assessment and Scoring Systems

The Hypophosphatemia Severity Score (HSS) incorporates serum phosphate level, presence of respiratory alkalosis, muscle weakness, and altered mental status to stratify patients into risk categories [21]. Patients scoring ≥6 points demonstrate significantly higher rates of mechanical ventilation failure and ICU mortality.

The Phosphate Depletion Syndrome Score (PDSS) emphasizes clinical manifestations over laboratory values, assigning points for hyperventilation, muscle weakness, rhabdomyolysis, hemolysis, and neurological symptoms [82]. This score better predicts clinical outcomes than phosphate levels alone, with scores > 4 associated with prolonged mechanical ventilation and increased mortality.

The Hypophosphatemia Risk Score, developed from mechanically ventilated patients, incorporates baseline phosphate level, APACHE II score, sepsis presence, nutritional status, and ventilator parameters [22]. Patients scoring >12 points demonstrated 85% probability of developing severe hypophosphatemia within 72 h.

### 5.4. Risk Stratification and Predictive Factors

The most consistent predictor of hyperventilation-induced hypophosphatemia is the degree of respiratory alkalosis. pH > 7.50 is associated with 75% incidence of hypophosphatemia, while PaCO_2_ levels below 25 mmHg correlate with severe depletion, particularly when sustained over 6 h [4]. Minute ventilation exceeding 12 L/min warrants close phosphate monitoring [15].

Patient-specific risk factors significantly influence susceptibility. Malnutrition, identified by BMI < 18.5 kg/m^2^, albumin < 3.0 g/dL, or recent weight loss >10%, increases risk threefold [36]. Chronic alcoholism, present in 15–20% of ICU admissions, depletes phosphate stores through poor intake, increased urinary losses, and vitamin D deficiency [83]. Additional risk factors include diabetic ketoacidosis, sepsis, major surgery, and prolonged fasting exceeding 7 days [82].

Refeeding syndrome risk assessment provides valuable insights. The NICE guidelines identify high-risk patients based on BMI < 16 kg/m^2^, unintentional weight loss >15% in 3–6 months, minimal nutritional intake for >10 days, or low baseline electrolytes [84].

The Refeeding Syndrome Risk Score additionally incorporates comorbidities and medication history, with scores > 11 predicting severe electrolyte abnormalities [85].

### 5.5. Diagnostic Algorithms and Dynamic Assessment

A systematic approach improves recognition. Initial screening should include phosphate measurement in all patients with unexplained tachypnea or difficult ventilator weaning [1]. If phosphate is below 2.0 mg/dL with concurrent respiratory alkalosis, additional testing should evaluate alternative causes including pulmonary embolism, sepsis, and anxiety disorders [15].

The phosphate repletion test provides diagnostic confirmation when uncertainty exists. Administration of 15–30 mmol intravenous phosphate over 4–6 h with resolution of hyperventilation within 24 h strongly supports the diagnosis [25].

Dynamic risk assessment using the phosphate trajectory index, calculated as percentage change over 6 h, identifies patients developing acute depletion [86]. A decline exceeding 25% within 6 h of hyperventilation onset predicts severe hypophosphatemia with 80% sensitivity and 75% specificity [86].

Integration of risk assessment into clinical protocols improves outcomes through prophylactic supplementation, pre-emptive repletion, and increased monitoring frequency in high-risk patients [12]. Health record-based alerts incorporating risk factors can prompt timely intervention, demonstrating 40% reduction in severe hypophosphatemia incidence [87,88]. Future directions include machine learning algorithms and personalized prediction models based on genetic polymorphisms affecting phosphate metabolism [14].

## 6. Impact on Clinical Outcomes: Impact on Mechanical Ventilation and Weaning Outcomes, Morbidity, Mortality

Hypophosphatemia significantly compromises liberation from mechanical ventilation and overall clinical outcomes in critically ill patients. The condition creates a complex cascade of physiological dysfunction that extends from respiratory muscle impairment to systemic organ failure, with impacts ranging from weaning failure to increased mortality.

### 6.1. Respiratory Muscle Function and Weaning Outcomes

The primary mechanism involves respiratory muscle weakness, particularly affecting the diaphragm, which exhibits reduced contractile force and endurance during phosphate depletion [5]. Phosphate depletion impairs respiratory muscle function through decreased ATP availability and altered calcium homeostasis, reducing diaphragmatic contractility by up to 50% [7]. Studies demonstrate that patients with serum phosphate levels below 2.0 mg/dL experience weaning failure rates of 30–45%, compared to 10–15% in patients with normal phosphate levels [15]. This creates a mismatch between ventilatory demand and capacity, manifesting as rapid shallow breathing index (RSBI) values exceeding 105 despite adequate gas exchange [89].

Hypophosphatemia profoundly affects spontaneous breathing trial (SBT) performance, with failure rates inversely correlating with phosphate levels. Patients with phosphate below 2.0 mg/dL demonstrate SBT failure rates of 60–70%, characterized by tachypnea, increased work of breathing, and hemodynamic instability within 30 min of trial initiation [20]. Successful phosphate repletion before SBT significantly improves outcomes, with normalized phosphate levels associated with 80% SBT success rates compared to 35% in persistently hypophosphatemic patients [17].

### 6.2. Duration of Mechanical Ventilation

Multiple observational studies document prolonged ventilation duration in hypophosphatemic patients. A multicenter cohort study of 1200 mechanically ventilated patients found that those with phosphate levels below 2.0 mg/dL required an average of 4.2 additional ventilator days compared to normophosphatemic controls [23]. The temporal relationship follows a dose–response pattern, with each 0.5 mg/dL decrease in serum phosphate associated with approximately 1.5 additional ventilator days [22]. Severe hypophosphatemia (<1.0 mg/dL) carries the worst prognosis, with median ventilation durations exceeding 14 days and mortality rates approaching 40% [90].

### 6.3. Mortality and Morbidity

Mortality rates correlate strongly with the severity and duration of hypophosphatemia. A multicenter cohort study of 2730 mechanically ventilated patients found 28-day mortality rates of 42% in severe hypophosphatemia versus 26% in normophosphatemic controls [22]. The association persists after adjustment for illness severity, with hypophosphatemia remaining an independent predictor of mortality (adjusted odds ratio 1.82, 95% CI 1.41–2.35) [23]. Patients developing hypophosphatemia within 24 h of initiating mechanical ventilation demonstrate worse outcomes than those with later onset [1].

### 6.4. Infectious and Cardiovascular Complications

Infectious complications occur more frequently in hypophosphatemic patients due to impaired immune function. Neutrophil chemotaxis, phagocytosis, and bacterial killing capacity decrease with phosphate depletion, while lymphocyte proliferation and antibody production are compromised [42]. Ventilator-associated pneumonia rates increase from 18% to 31% in patients with sustained hypophosphatemia, with longer antibiotic requirements and higher rates of multidrug-resistant organisms [24].

Cardiovascular morbidity includes arrhythmias, heart failure, and hemodynamic instability. Severe hypophosphatemia reduces myocardial contractility and cardiac output, necessitating increased vasopressor support [52]. A prospective study found new-onset atrial fibrillation in 28% of hypophosphatemic patients versus 12% of controls, with ventricular arrhythmias occurring in 8% versus 2%, respectively [91]. These cardiac complications contribute to prolonged ICU stays and increased resource utilization.

### 6.5. Functional Recovery and Economic Impact

Neuromuscular weakness extends beyond respiratory muscles, affecting overall functional recovery. ICU-acquired weakness develops in 60% of patients with severe hypophosphatemia compared to 35% of those maintaining normal levels [8]. This weakness persists after ICU discharge, with reduced six-month functional independence scores and delayed return to baseline activities [37].

The economic burden is substantial, with hypophosphatemia-associated weaning failure increasing ICU costs by approximately $45,000 per patient [37]. Mean ICU length of stay extends from 7.2 to 11.8 days, while hospital stays increase from 18 to 27 days [21]. Total hospitalization costs rise by approximately 40%, driven by prolonged mechanical ventilation, increased medication requirements, and management of complications [92].

### 6.6. Treatment Implications

The temporal relationship between hypophosphatemia correction and outcome improvement supports causality. Early phosphate repletion within 24 h of detection is associated with reduced ventilator days, lower infection rates, and improved survival [81]. However, the optimal repletion strategy remains uncertain, with both under-treatment and aggressive replacement carrying risks [12]. Understanding these outcome relationships guides clinical decision-making and emphasizes the importance of preventing hyperventilation-induced hypophosphatemia in critically ill patients.

## 7. Management Strategies: From Phosphate Repletion to Ventilatory Support

Management of hyperventilation-induced hypophosphatemia requires a systematic approach addressing both respiratory alkalosis and phosphate depletion. The primary intervention involves correcting the underlying respiratory alkalosis, when possible, though this may not be feasible in all critically ill patients, making concurrent phosphate replacement essential [12]. Prevention strategies focus on identifying high-risk patients, with prophylactic phosphate supplementation of 15–30 mmol daily reducing severe hypophosphatemia incidence by 60% in mechanically ventilated patients anticipated to require high minute ventilation [93]. Careful ventilator management targeting the lowest minute ventilation compatible with adequate gas exchange, as well as maintaining PaCO_2_ between 35–45 mmHg when permissive hypercapnia is tolerated, significantly reduces hypophosphatemia risk [5,7].

### 7.1. Phosphate Repletion Protocols by Severity

For mild hypophosphatemia (2.0–2.5 mg/dL) in asymptomatic patients, oral supplementation with 30–60 mmol daily in divided doses usually suffices, with gradual replacement over 3–5 days minimizing adverse effects while effectively repleting stores [26].

Moderate hypophosphatemia (1.0–2.0 mg/dL) typically requires intravenous replacement with sodium or potassium phosphate at doses of 0.16–0.32 mmol/kg over 4–6 h. Standard protocols recommend 15–30 mmol infused over 4–6 h, with serum phosphate rechecked 2 h after completion [25].

A weight-based protocol implementing 15 mmol phosphate for patients < 60 kg, 20 mmol for 60–80 kg, and 30 mmol for >80 kg achieves target levels in 85% of patients within 24 h [94].

Severe hypophosphatemia (<1.0 mg/dL) necessitates aggressive replacement with 0.5–0.64 mmol/kg over 6–8 h or 30–60 mmol over 6–8 h, with some protocols advocating continuous infusions of 7.5 mmol/h until levels exceed 1.0 mg/dL [26,95,96]. The maximum safe infusion rate remains 7.5 mmol/h to avoid metastatic calcification and acute hypocalcemia [97]. Continuous infusion protocols demonstrate superior efficacy compared to bolus administration, maintaining steady-state levels and reducing rebound hypophosphatemia [93].

### 7.2. Monitoring and Safety Considerations

Successful phosphate repletion requires vigilant monitoring with serum phosphate levels rechecked 2–4 h after infusion completion [12]. Concurrent measurement of calcium, magnesium, and potassium is essential, as phosphate administration can precipitate hypocalcemia through calcium-phosphate complex formation [95,97]. Renal function assessment before repletion prevents phosphate accumulation in patients with acute kidney injury.

Patients receiving doses exceeding 0.5 mmol/kg require cardiac monitoring and serial calcium measurements every 4 h during infusion [98]. Electrocardiographic monitoring during rapid replacement helps detect arrhythmias from electrolyte shifts [95]. Documentation of baseline parathyroid hormone levels helps differentiate primary phosphate disorders from redistribution phenomena.

### 7.3. Ventilatory Management Strategies

While correcting hypophosphatemia, pressure support ventilation with levels of 10–15 cm H_2_O compensates for respiratory muscle weakness while maintaining spontaneous breathing efforts [7]. Avoiding excessive sedation prevents further respiratory depression and allows accurate assessment of intrinsic respiratory drive [15]. The hyperventilation typically resolves within 24–48 h of achieving phosphate levels above 2.5 mg/dL [20]. During this transition, permissive hypocapnia (PaCO_2_ 30–35 mmHg) prevents patient–ventilator dyssynchrony and reduces work of breathing [51].

Daily spontaneous breathing trials should commence only after phosphate levels exceed 2.0 mg/dL for at least 24 h [16]. The combination of normalized phosphate levels, resolved hyperventilation, and adequate respiratory muscle strength predicts successful liberation from mechanical ventilation in 90% of cases [99].

### 7.4. Special Populations and Integrated Approach

Patients receiving continuous renal replacement therapy require higher replacement doses of 10–15 mmol per liter of effluent due to ongoing losses [18]. Those with renal dysfunction need reduced doses and extended infusion times to prevent hyperphosphatemia. Malnourished patients at risk for refeeding syndrome benefit from preemptive supplementation before initiating nutrition support [84]. Nutritional support should provide 10–15 mmol phosphate per 1000 kcal to prevent refeeding-associated depletion [36].

Recent evidence supports individualized replacement strategies based on total body deficit estimation rather than serum levels alone, with phosphate deficit calculations incorporating body weight, distribution volume, and target levels improving replacement accuracy [100]. Multidisciplinary protocols incorporating nursing, pharmacy, and physician input improve compliance and reduce complications [101]. This integrated approach, combining aggressive phosphate repletion with supportive ventilatory management, significantly reduces ventilation duration and improves clinical outcomes.

## 8. Future Perspectives: Biomarkers, Monitoring Technologies, and Precision Medicine Approaches

Current limitations of serum phosphate measurements in reflecting total body stores have catalyzed research into superior biomarkers. Fibroblast growth factor 23 (FGF23), a phosphaturic hormone, emerges as a promising early indicator, with levels rising before serum phosphate falls [102]. Recent studies demonstrate that FGF23 levels above 100 RU/mL predict hypophosphatemia development within 48 h with 85% sensitivity and 78% specificity [103,104].

Urinary phosphate-to-creatinine ratio and fractional excretion of phosphate provide non-invasive assessments of renal phosphate handling, potentially distinguishing between redistribution and true depletion while identifying at-risk patients [105]. Red blood cell phosphate content correlates more closely with intracellular stores than serum levels, offering a practical monitoring alternative [106]. Emerging proteomic approaches have identified phosphate-responsive proteins including osteopontin and matrix Gla protein as potential biomarkers for phosphate depletion severity [107].

Metabolomic profiling reveals distinct depletion patterns, with nuclear magnetic resonance spectroscopy identifying alterations in phosphometabolite profiles, including decreased ATP and phosphocreatine levels, before clinical manifestations appear [10]. Additionally, microRNA expression patterns, particularly miR-223 and miR-146a, correlate with cellular phosphate status and inflammatory responses, offering diagnostic and prognostic value [108].

### 8.1. Advanced Monitoring Technologies

Continuous phosphate monitoring represents a critical unmet need in intensive care. Point-of-care devices utilizing ion-selective electrodes show promise for real-time measurement, enabling dynamic tracking of phosphate fluctuations during critical illness similar to continuous glucose monitoring systems [109]. Microdialysis techniques enabling tissue-level phosphate assessment have demonstrated feasibility in animal models, revealing organ-specific depletion patterns not reflected in serum measurements [110].

Near-infrared spectroscopy adapted for phosphate detection offers non-invasive monitoring potential, with preliminary studies showing correlation between spectroscopic signatures and serum phosphate levels [111]. Integration of continuous phosphate monitoring with ventilator parameters could enable automated detection of hypophosphatemia-induced hyperventilation, triggering alerts for timely intervention [112]. Wearable sensors incorporating phosphate-sensitive materials are under development for continuous monitoring in less acute settings [113].

### 8.2. Precision Medicine and Genetic Approaches

Genetic variations significantly influence individual susceptibility to hypophosphatemia. Polymorphisms in SLC34A1 and SLC34A3 genes encoding renal phosphate transporters affect baseline phosphate levels and response to supplementation [114]. Pharmacogenomic studies reveal that variants in FGF23 and KLOTHO genes modulate phosphate homeostasis during critical illness, suggesting potential for personalized repletion protocols [115].

Machine learning algorithms demonstrate remarkable predictive capacity, analyzing electronic health record data to predict hypophosphatemia development with 92% accuracy by integrating clinical variables, medications, and laboratory trends [116]. These predictive models enable preemptive supplementation in high-risk patients, potentially preventing ventilatory complications [117]. Artificial intelligence-driven decision support systems incorporating patient-specific factors optimize phosphate repletion dosing and monitoring frequency [17].

### 8.3. Therapeutic Innovations

Novel phosphate formulations promise improved bioavailability and reduced adverse effects. Liposomal phosphate preparations demonstrate enhanced cellular uptake and sustained release properties in preclinical studies [118]. Targeted delivery systems utilizing phosphate-loaded nanoparticles and nanomedicine approaches with pH-sensitive release properties show promise for organ-specific repletion while minimizing systemic complications [119,120].

Research into phosphate-sensing pathways has identified therapeutic targets beyond simple repletion. Modulation of sodium-phosphate cotransporter expression through small molecules could enhance endogenous phosphate retention [121]. FGF23 antagonists under investigation for chronic kidney disease may prevent excessive phosphate wasting in acute settings [122,123].

Cellular therapies represent a frontier approach, with mesenchymal stem cells modified to express high levels of phosphate transporters showing promise in animal models for rapidly correcting intracellular deficits [124]. Gene therapy targeting NaPi-II transporter expression could provide long-term solutions for patients with recurrent hypophosphatemia, though safety evaluation remains essential [107].

## 9. Conclusions and Clinical Recommendations

The intricate relationship between hypophosphatemia and hyperventilation represents a critical yet often overlooked aspect of respiratory management in intensive care. The intricate relationship between phosphate depletion and respiratory function affects multiple levels of control, from cellular energy metabolism to central chemoreceptor sensitivity, ultimately compromising both spontaneous breathing and mechanical ventilation outcomes [125]. With prevalence reaching 80% in certain critically ill populations, this condition demands systematic screening and management protocols [1].

In terms of hyperventilation-induced hypophosphatemia, the evidence demonstrates that acute respiratory alkalosis triggers rapid intracellular phosphate shifts, leading to potentially severe serum depletion with serious clinical consequences [6]. Clinical manifestations extend beyond simple electrolyte imbalance to encompass respiratory muscle dysfunction, altered ventilatory drive, and impaired tissue oxygen delivery, synergistically contributing to prolonged mechanical ventilation, increased weaning failure rates, and higher mortality [23]. Importantly, serum phosphate levels poorly reflect total body stores, emphasizing the need for clinical vigilance and consideration of cellular depletion even with borderline serum values [6].

### 9.1. Clinical Recommendations and Protocol Development

Systematic phosphate monitoring should be implemented for all mechanically ventilated patients, particularly those with minute ventilation exceeding 12 L/min or pH > 7.50 [1]. All mechanically ventilated patients should undergo phosphate assessment within 6 h of ICU admission, with daily monitoring during the acute phase of illness [3]. High-risk patients, including those with malnutrition, alcoholism, diabetic ketoacidosis, sepsis, or refeeding risk, warrant prophylactic supplementation and increased monitoring frequency, with risk stratification tools guiding monitoring frequency and repletion aggressiveness [12,126].

Standardized repletion protocols based on severity stratification improve outcomes while minimizing complications. Moderate hypophosphatemia (1.0–2.0 mg/dL) warrants intravenous replacement with 0.16–0.32 mmol/kg, while severe depletion (<1.0 mg/dL) requires 0.5–0.64 mmol/kg with intensive monitoring [12]. When hypophosphatemia develops, intravenous supplementation is preferred for levels below 2.0 mg/dL [127]. Close monitoring of calcium, magnesium, and potassium during replacement remains essential to prevent complications.

Ventilator management should prioritize prevention through careful control, targeting the lowest minute ventilation compatible with adequate gas exchange. During hypophosphatemia, appropriate support levels should accommodate increased respiratory drive while avoiding excessive sedation that masks underlying metabolic derangements [7].

### 9.2. Quality Improvement and Implementation Strategies

Implementation requires coordinated quality improvement efforts with electronic health record integration. Automated alerts for hypophosphatemia and trending capabilities enhance detection and treatment compliance [2].

Educational initiatives targeting respiratory therapists, intensivists, and nursing staff improve recognition of hypophosphatemia-related ventilatory abnormalities. Simulation-based training incorporating hypophosphatemia scenarios enhances clinical pattern recognition and management skills [128]. Regular audits of phosphate monitoring compliance and clinical outcomes drive continuous improvement in protocol implementation [129]. Institutional protocols incorporating multidisciplinary input optimize care delivery and reduce practice variability.

### 9.3. Future Research Priorities and Knowledge Gaps

The most critical limitation in current hypophosphatemia management is the complete absence of randomized controlled trials evaluating phosphate replacement strategies and their impact on respiratory outcomes. This evidence gap severely limits our ability to make strong clinical recommendations. Urgent priorities include conducting well-designed RCTs to establish optimal phosphate replacement protocols, target levels for ventilator liberation, and timing of supplementation in mechanically ventilated patients.

Beyond addressing this fundamental gap, prospective trials should evaluate validated risk prediction tools [3,130] and develop continuous monitoring technologies for real-time titration [90]. Research into intracellular phosphate biomarkers could improve identification of patients requiring aggressive repletion despite normal serum levels [43]. Integration of precision medicine approaches may enable individualized strategies.

Until robust RCT evidence becomes available, clinicians must rely on observational data and expert consensus. Despite these limitations, maintaining awareness of hyperventilation-induced hypophosphatemia’s impact on weaning success and mortality remains crucial, as early recognition based on current best evidence significantly improves outcomes [23]. Implementing evidence-based strategies, though derived from lower-quality evidence, remains necessary to minimize this complication’s impact and improve survival and functional recovery.

## Figures and Tables

**Table 1:** Clinical Manifestations of Hypophosphatemia–Hyperventilation Syndrome.

System/Category	Clinical Manifestations	Specific Features	Phosphate Threshold	References
Respiratory	Hypophosphatemia-induced hyperventilation	Unexplained tachypnea (25–40 breaths/min) Increased minute ventilation Rapid, shallow breathing pattern Compensated respiratory alkalosis (pH 7.45–7.55, PaCO_2_ 20–30 mmHg) Normal arterial oxygen saturation Absence of perioral paresthesias/carpopedal spasm	<1.5 mg/dL	[4,9,20,68]
	Hyperventilation-induced respiratory muscle weakness	Diaphragmatic dysfunction Decreased maximal inspiratory pressure Reduced transdiaphragmatic pressure Impaired hypercapnic drive response 50% develop proximal muscle weakness Impaired cough effort	Variable	[7,8]
Neurological	Central nervous system	Altered mental status (irritability → confusion → delirium → coma) Seizures (rare) Central pontine myelinolysis (severe cases) Often precedes respiratory changes	<1.5 mg/dL	[69,70]
	Peripheral nervous system	Paresthesias Hyperesthesia Tremor Peripheral neuropathy	<1.5 mg/dL	[70]
Neuromuscular	Muscle dysfunction	Rhabdomyolysis (elevated CK, myoglobinuria) Proximal myopathy Difficulty with ambulation General muscle weakness	<0.5 mg/dL (rhabdomyolysis)	[9,60]
Hematological	Red blood cell abnormalities	Hemolytic anemia Decreased erythrocyte ATP 2,3-DPG depletion Increased RBC rigidity Splenic sequestration	<1.0 mg/dL	[71]
	White blood cell dysfunction	Impaired chemotaxis Decreased phagocytosis Reduced bactericidal activity Increased infection susceptibility	<1.0 mg/dL	[45]
	Platelet dysfunction	Prolonged bleeding time Clinically significant bleeding (rare)	<1.0 mg/dL	[42]
Cardiac	Myocardial effects	Reversible myocardial depression Decreased contractility Reduced cardiac output Congestive heart failure (with underlying disease) Ventricular arrhythmias (rare)	<1.0 mg/dL	[52,72]
	ECG changes	ST-segment depression T-wave flattening QT prolongation	<1.0 mg/dL	[72]
Metabolic	Glucose metabolism	Insulin resistance Impaired glucose metabolism Decreased ATP for insulin signaling	<1.5 mg/dL	[49]
	Acid-base disturbance	Paradoxical metabolic acidosis Impaired renal acid excretion Increased organic acid production	<1.0 mg/dL	[50]
Hepatic (Acute Liver Failure)	Liver-specific manifestations	Occurs in 45–65% of ALF patients Biphasic pattern: initial release then depletion Impaired hepatic regeneration Decreased vitamin D conversion Mortality > 80% when PO_4_ < 0.5 mg/dL	<1.0 mg/dL (severe) <0.5 mg/dL (critical)	[73,74]

Severe/Life-threatening. PO_4_ = Serum Phosphate. ALF = Acute Liver Failure. ABG = Arterial Blood Gas.

## Data Availability

No new data were created for this article.

## Abbreviations

The following abbreviations are used in this manuscript:

ICU	Intensive care unit
FGF23	Fibroblast growth factor 23
2,3-DPG	2,3-diphosphoglycerate
